# Anti-Inflammatory and Anti-Catabolic Effects of Creatine Supplementation: A Brief Review

**DOI:** 10.3390/nu14030544

**Published:** 2022-01-27

**Authors:** Dean M. Cordingley, Stephen M. Cornish, Darren G. Candow

**Affiliations:** 1 Applied Health Sciences, University of Manitoba, Winnipeg, MB R3T 2N2, Canada; umcordid@myumanitoba.ca (D.M.C.); Stephen.Cornish@umanitoba.ca (S.M.C.); 2 Pan Am Clinic Foundation, 75 Poseidon Bay, Winnipeg, MB R3M 3E4, Canada; 3 Faculty of Kinesiology and Recreation Management, University of Manitoba, Winnipeg, MB R3T 2N2, Canada; 4 Centre for Aging, University of Manitoba, Winnipeg, MB R3T 2N2, Canada; 5 Faculty of Kinesiology and Health Studies, University of Regina, Regina, SK S4S 0A2, Canada

**Keywords:** cytokines, cancer, oxidative stress, muscle protein, bone catabolism

## Abstract

It is well established that creatine supplementation, primarily when combined with resistance training, significantly increases measures of muscle mass and performance (primarily strength). Emerging research also indicates that creatine supplementation may have favorable effects on measures of bone biology. These anabolic adaptations may be related to creatine influencing cellular hydration status, high-energy phosphate metabolism, growth factors, muscle protein kinetics, and the bone remodeling process. Accumulating research also suggests that creatine supplementation has anti-inflammatory and anti-catabolic properties, which may help create a favorable environment for muscle and bone accretion and recovery from exercise. Creatine supplementation has the ability to decrease markers of inflammation and possibly attenuate cancerous tumor growth progression. From a musculoskeletal perspective, there is some evidence to show that creatine supplementation reduces measures of muscle protein catabolism (primarily in males) and bone resorption when combined with resistance training. The purpose of this brief review is to summarize the current body of literature examining the potential anti-inflammatory and anti-catabolic effects of creatine supplementation across various research populations.

## 1. Introduction

Since the seminal study by Harris et al. in 1992 [1], research investigating the multi-factorial effects of creatine supplementation has substantially increased. The current body of research indicates that creatine supplementation (typically when combined with resistance training) preferentially increases measures of muscle mass and performance (for meta-analysis reviews [2,3,4,5]) and may be of particular benefit for the elderly to counteract age-related muscle atrophy and sarcopenia [6]. There is also preliminary research showing that creatine supplementation and resistance training have better effects on measures of bone biology [7,8]. Mechanistically, from an anabolic muscle perspective, creatine supplementation has been shown to increase cellular hydration status, intramuscular creatine stores (primarily phosphocreatine), myogenic transcription factors and satellite cell activity, insulin-like growth-factor 1, and protein kinases downstream in the mammalian target of rapamycin (mTOR), which are involved in protein translation (For reviews see [4,7,8]). Furthermore, from an anabolic bone perspective, creatine stimulates creatine kinase and osteoblast cell activity (cells involved in bone formation) [8]. Alternatively, increased muscle accretion from creatine may act as a pulley and bone as a lever during muscle contractions resulting in osteocyte-induced bone formation [9]. In addition to these purported anabolic mechanisms and processes, there is a small body of research suggesting that creatine supplementation has anti-inflammatory and anti-catabolic effects on properties of muscle and bone biology, which may help create a more favorable environment for muscle and bone growth or recovery from exercise over time. Decreasing inflammation is important because it has a negative effect on muscle protein kinetics, which is a hallmark characteristic of sarcopenia or the age-related reduction in muscle mass and function in older adults [10]. Subsequently, sarcopenia is associated with reduced bone mass in older adults [7,10]. Therefore, the purpose of this brief review is to summarize the current body of research investigating the anti-inflammatory and anti-catabolic effects of creatine supplementation across various research populations.

## 2. Anti-Inflammatory Effects of Creatine Supplementation

Acutely, inflammation supports the immune response to trauma or infection. However, chronic low-grade inflammation can disrupt the function of physiological systems throughout the body [11]. Human aging is associated with elevated low-grade inflammation [12], and leads to detrimental effects on skeletal muscle [13] and bone [14], and contributes to age-related cognitive decline [15]. Aside from aging, chronic inflammation is associated with a variety of diseases and medical conditions, including cancer [16], asthma [17], osteoarthritis [18], heart failure [19], inflammatory bowel diseases [20], along with others [21]. It has been postulated that creatine supplementation could beneficially moderate the inflammatory response in the setting of aging [8] and disease [22,23].

There is some research that has evaluated the effects of creatine supplementation in a variety of models of inflammation [24,25,26,27,28,29,30,31,32,33,34,35,36,37,38,39,40], demonstrating a benefit. However, other research demonstrates no advantage or a detriment to inflammation. The purpose of this section is to outline the current evidence for creatine supplementation in a variety of inflammatory models.

The first study to evaluate the effects of creatine on inflammation used a rat model to induce inflammation in the paw by carrageenan injection, which activates the cyclo-oxygenase enzymatic pathway of inflammation [24]. In this study, the authors found that intraperitoneal injection of creatine was able to decrease edema in the rat paws [24]. Further research from the same group found that orally administering creatine to rats was able to reduce carrageenan injection-induced inflammation of the paw to the same degree as a non-steroidal anti-inflammatory drug (NSAID) used in treating animals and that the creatine supplementation had similar analgesic effects to the NSAID [26]. Research from this same group found that creatinine (the by-product of creatine metabolism) was able to reduce edema in a variety of rat models of inflammation, including a type of arthritis induced by formaldehyde [25]. These studies were the first line of evidence that creatine, or its by-product creatinine, may have anti-inflammatory properties associated with them. 

The possible anti-inflammatory effects of creatine have been evaluated in models of disease associated with inflammation such as cancer [38,41]. Here, the researchers gave rats with tumor cells creatine (*n* = 10; 300 mg⋅kg^−1^⋅day^−1^) or placebo (*n* = 9; water) via intragastric gavage and compared this to a control condition (*n* = 6). The results indicated that systemic plasma interleukin-6 (IL-6; a pleiotropic cytokine) was lower and IL-10 (an anti-inflammatory cytokine) was higher in the rats receiving the creatine (assessed by LUMINEX^®^ magnetic multi-bead assay), which the authors suggested was evidence of a stronger anti-inflammatory environment [38]. Further, the growth of the tumor, which was determined through caliper measurement, was slowed in the creatine supplemented group when compared to the placebo, providing some initial evidence that creatine may be effective in dealing with malignancies. Supporting these findings, Di Biase et al. in 2019 [42] identified that supplementing mice with creatine (10.5 mg daily for injection based administration and 3 g⋅kg^−1^⋅day^−1^ for diet based administration) slowed tumor growth determined by measurement with digital calipers (*n* = 3–4), and that creatine was an important metabolic regulator of antitumor CD8 T cell immunity (*n* = 3–9) quantified with quantitative polymerase chain reaction for creatine transporter, flow cytometry analysis or western blot analysis. Taken together, it appears that creatine has the potential to suppress tumor growth and improve T cell-based immunotherapies for cancer treatment [38,41,42]. 

An in vitro study of human pulmonary endothelial cells (*n* = 5) demonstrated that 5 mM of creatine was able to suppress the adhesion of neutrophils to the endothelial cells (determined by the ratio of radioactively adherent neutrophils by the total neutrophils); and creatine at doses of 0.5 mM or greater induced inhibition of intracellular adhesion molecule-1 (ICAM-1) and E-selectin (radioactive immunochemical assay) on the endothelial cells, which suggested anti-inflammatory activity of creatine in this model [27]. However, in a mouse model of airway inflammation (asthma) (creatine, *n* = 8; placebo, *n* = 8), creatine supplementation (0.5 g⋅kg^−1^⋅day^−1^, 5 days per week) for 4-weeks did not reduce airway inflammation or inflammatory mediators, whereas pro-inflammatory mediators were up-regulated (determined by immunohistochemistry and morphometry) with creatine supplementation, suggesting inflammatory asthma could be exacerbated [31,34]. In a rat model of lung ischemia/reperfusion, five days of creatine supplementation (0.5 g⋅kg^−1^⋅day^−1^; *n* = 10) was able to reduce acute lung injury through anti-inflammatory mechanisms, which included an attenuation of Toll-like receptor 4 (TLR-4; a signal for the activation of nuclear factor kappa B [NF-κB] and initiation of the innate immune system inflammatory response) measured with Western blotting [39]. Thus, it seems that creatine supplementation may be effective at lowering inflammation in some animal models of lung injury but may exacerbate the inflammatory response in others. 

Pre-clinical mouse data (*n* = 95) suggests that seven days of creatine supplementation (6.25 g⋅kg^−1^·day^−1^) can reduce nociception induced by inflammation (30 μL injection of 4% formalin solution to a hind paw) determined by the time spent licking the injected paw, which may be mediated through activation of acid-sensing ion channels (ASICs) [43]. More research is needed to fully understand the complexity of creatine supplementation protocols on acute and long-term inflammatory responses in various diseases. Histological evaluation of mouse (*n* = 18) and rat models (*n* = 12) of long-term creatine supplementation (0.025 ± 0.05 g⋅kg^−1^⋅day^−1^ 300 days and 2% *wt*/*wt* diet for 365 days respectively) revealed that in the mouse model there were significant hepatic inflammatory lesions noted, whereas the rat model did not demonstrate the same hepatic inflammatory lesions [28]. The results of the study also indicated no other tissues evaluated demonstrated inflammatory lesions of any type in either the mouse or rat model [28]. Therefore, it appears that creatine supplementation may have species-specific effects on inflammatory processes and could be tissue-specific in certain species (mice). While the effects of creatine as an anti-inflammatory have been reported in animal models, we will now turn our attention to the effects that creatine may have in humans in modulating the inflammatory response due to diseased states.

Given the potential of creatine supplementation to reduce inflammation, its use has been examined in a variety of human diseased states. Cornish and Peeler, in 2018, examined the effects of a 12-week creatine (*n* = 9) versus placebo (*n* = 9) supplementation protocol on inflammatory biomarkers in individuals diagnosed with knee osteoarthritis [40]. Knee osteoarthritis is characterized by a low level of inflammation that may be responsible for the progressive destruction of the joint and an exacerbation of the symptoms. The authors hypothesized that creatine supplementation (20 g∙d^−1^ for 1 week and then 5 g∙d^−1^ for 11 weeks) would lower systemic biomarkers of inflammation in the knee osteoarthritis participants when compared to the placebo. However, none of the biomarkers assessed with enzyme-linked immunosorbent assay (ELISA) (C-reactive protein (CRP), IL-1β, IL-6, s100 A8/A9, and tumor necrosis factor-alpha (TNF-α)) were significantly affected by the creatine supplementation protocol [40]. In a study that evaluated the combined effect of aerobic exercise training (3 d∙wk^−1^) with creatine supplementation (5 g∙d^−1^; *n* = 50) for 8 weeks compared to no intervention (*n* = 50) in heart failure patients, the combined intervention resulted in decreased systemic IL-6 and CRP as well as ICAM-1 and P-selectin (which are markers of endothelial dysfunction that were measured by ELISA) [44]. Even though there was a significant decrease of these biomarkers in heart failure patients, it is unknown if the creatine supplementation protocol was the cause of this effect as the intervention was combined with aerobic exercise. It is well known that aerobic exercise training can have a positive effect on inflammatory mediators [45], and thus, it seems necessary to evaluate both aerobic exercise and creatine supplementation on their own when assessing changes in systemic inflammation over time. It has been suggested that creatine supplementation could be of value in other disease states, which currently lack clinical data (such as inflammatory bowel diseases) because of its postulated ability to modulate the immune response and reduce inflammatory induced pain [46].

Aside from models of disease, the anti-inflammatory effects of creatine have been investigated in exercise-induced inflammation. Research in a rat model of eccentric exercise (downhill running to exhaustion) had animals supplement with creatine (*n* = 12; 300 mg∙kg^−1^∙d^−1^) or saline (*n* = 12) by gavage for 15 days before sacrificing them 24 or 48 h following the eccentric exercise [36]. The results showed no effect of creatine supplementation on attenuating the skeletal muscle protein levels of TNF-α, IL-1β, or NF-κB (determined with immunoblotting) increases when compared to saline control [36].

Several studies evaluated the effects of creatine supplementation on a variety of systemic inflammatory mediators after an acute bout of various types of exercise in humans [29,30,32,37]. Santos and colleagues found that 5 days of creatine supplementation (creatine, *n* = 18; placebo, *n* = 16) (20 g∙d^−1^) attenuated prostaglandin-E2 (PGE2) and TNF-α (determined with ELISA kits) increases associated with running 30 km at race pace in healthy young active men [29]. The study also found creatine attenuated increases in creatine kinase (an indirect marker of skeletal muscle damage; quantified via spectrophotometry) and eliminated any increase in lactate dehydrogenase (another marker of muscle damage; quantified via spectrophotometry) when compared to the placebo group [29]. Supporting this previous work, Bassit et al. demonstrated that triathletes completing a half-ironman triathlon who supplemented with creatine (20 g⋅day^−1^; *n* = 5) for five days prior to the race experienced an attenuation of TNF-α, interferon-α (IFN-α), IL-1β, and PGE2 (all analyzed with commercially available ELISA kits) 24–48 h post-race when compared to placebo (*n* = 6) [32]. These two studies provided evidence that creatine supplementation may reduce the inflammatory response to acute endurance/aerobic-based exercise. However, in a small human study examining the effects of short term creatine supplementation (5 days × 20 g∙d^−1^; *n* = 4) or placebo (*n* = 4) on systemic CRP concentrations in triathletes completing an ironman triathlon, the researchers found no effect of the creatine on attenuating the CRP (determined spectrophotometrically with a commercially available kit) response 36 and 60 h following the race when compared to placebo [33]. Research evaluating the effects of creatine supplementation on inflammatory markers in anaerobic and resistance exercise generated some contrasting results [30,37]. Deminice et al. recruited 25 younger (Age: 17.4 ± 1.2 years) soccer players who were randomized to either creatine supplementation (0.3 g∙kg^−1^) or placebo group and subjected to a repeated anaerobic sprint test (6 × 35 m running sprints with 10 s of rest between sprints) before and after 7 days of supplementation. The results indicated that increases in TNF-α and CRP (both determined with commercially available competitive immunoassay kits) were attenuated in the creatine supplemented group when compared to placebo [37], suggesting a dampening effect of creatine on the inflammatory response to intense sprinting exercise. However, Rawson et al. found that there was no effect of 10 days of creatine supplementation (0.3 g∙kg^−1^; *n* = 11) versus placebo (*n* = 11) on CRP concentration (measured with a high-sensitivity enzyme immunoassay kit) in the systemic circulation following acute resistance exercise (5 sets of 15–20 repetitions at 50% of 1-repetition maximum for the back squat exercise) in healthy resistance-trained men [30]. Similarly, the effects of creatine supplementation (5 g∙day^−1^; *n* = 16) during a 12-week resistance training program in older adults did not find any differences in IL-6, IL-10, CRP or monocyte chemoattractant protein-1 (MCP-1) (all measured by ELISA except for CRP, which was determined by turbidimetric assay) compared to placebo (*n* = 16), but did identify a decrease in MCP-1 following the resistance training program regardless of creatine consumption [47]. These contrasting results may be due to the type of exercise that was employed in each of the studies. 

More recently, studies have evaluated the efficacy of a combined nutritional supplement beverage intake to act in an anti-inflammatory manner. Bell and colleagues demonstrated the effectiveness of a multi-ingredient nutritional supplement (that contained creatine at a dose of 5 g∙d^−1^; *n* = 25) to lower systemic inflammatory markers including TNF-α and IL-6 (both measured with a Bio-Plex system) when compared to the control condition (*n* = 24) in healthy older men [48]. While this research is important, it fails to answer which of the ingredients used in the multi-ingredient supplement was able to lower the biomarkers of inflammation measured. Other ingredients included whey protein, vitamin-D, and ω-3 fatty acids, which all may have anti-inflammatory properties associated with them. Another study that evaluated the effects of whey protein and ω-3 fatty acid supplementation, with (*n* = 10) and without (*n* = 8) creatine (5 g∙d^−1^), in healthy young females (control, *n* = 10) who completed 5 high volume resistance exercise sessions on consecutive days to induce muscle damage and inflammation found no effect of either supplement protocol on systemic CRP and IL-6 concentrations (both determined by ELISA) [49]. A limitation of this study was that they only assessed the biomarkers at baseline and every 24 h after baseline immediately prior to the next training session; this would not allow for the acute measurement of these biomarkers immediately after the exercise session. Nonetheless, the addition of creatine to the nutritional supplement had no effect on the inflammatory markers measured [49]. Even though research on multi-ingredient supplements may be needed, it does not substantiate which supplements are most beneficial for lowering inflammation. Future research should identify which type of nutritional supplement is more effective for lowering inflammation and enhancing health or performance outcomes.

Current evidence on the effectiveness of creatine to lower various markers of inflammation in a variety of in vivo models is mixed and may be species and model-specific. Further research on the effects of creatine supplementation should likely focus on mechanistic studies in cell culture to identify if creatine has an effect in dampening the inflammatory response. Specifically, studies evaluating if the canonical pathway of NF-κB, which is stimulated by pro-inflammatory mediators such as TNF-α, IL-1β, and various toll-like receptors (TLR), can be modulated by creatine is an area of interest. Certainly, some research in a murine macrophage cell line has already demonstrated the effectiveness of creatine in downregulating a variety of TLRs, which may also result in immunosuppression via the innate immune system and a dampening of the NF-κB pathway [35]. If immunosuppression happens in creatine supplemented individuals, this may have implications for treating a variety of pathologies associated with the inflammatory response (i.e., creatine could be a viable treatment strategy with inflammatory pathologies). Still, it may also increase the incidence of opportunistic infections in healthy individuals if creatine is, in fact, immunosuppressive. More research will be required to elucidate the effects of creatine supplementation on inflammatory processes in a variety of pathologies as well as in healthy individuals. 

## 3. Anti-Catabolic Effects of Creatine Supplementation

For muscle, measures of muscle protein catabolism were primarily used to evaluate the anti-catabolic efficacy of creatine supplementation. To date, only 5 studies have examined the effects of creatine supplementation on measures of muscle protein catabolism. One study incorporated leucine infusion methodology while the remaining 4 studies used urinary 3-methylhistidine (3-MH), a surrogate measure of muscle protein catabolism. In young healthy males, creatine supplementation (20 g∙d^−1^ for 5 d followed by 5 g∙d^−1^ for a subsequent 3–4 d; *n* = 7) significantly reduced leucine oxidation by ~20% and plasma leucine rate of appearance by ~8% (indicator of muscle protein catabolism; quantified using gas chromatography-combustion-isotope ratio mass spectrometry of muscle biopsies from the vastus lateralis muscle) compared to those on placebo (*n* = 4). However, creatine had no effect on leucine oxidation or plasma leucine rate of appearance in females (creatine, *n* = 7; placebo, *n* = 4). The authors concluded that short-term creatine supplementation may provide anti-catabolic actions in lean tissue in males only [50]. Furthermore, young adults (males and females combined) who supplemented with creatine (9 g∙d^−1^; *n* = 47) during 5 weeks of intense resistance training (6 d∙wk) experienced a decrease in 3-MH (−0.4 to −4.7%; analyzed with high-performance liquid chromatography of 24 h urine samples) over time [51]. However, no sex analysis regarding differences in 3-MH was performed. In older males, creatine supplementation (0.1 g∙kg∙d^−1^ or ~9 g∙d^−1^; *n* = 23) during 10 weeks of supervised, whole-body resistance training, significantly reduced 3-MH (analyzed with high-performance liquid chromatography of 24 h urine samples) by 40% compared to a 29% increase for those on placebo (*n* = 12) [52]. Similarly, older males supplementing with creatine (0.1 g∙kg∙d^−1^ or ~9 g∙d^−1^; *n* = 7) during 12 weeks of drop-set resistance training experienced a significant decrease in 3-MH (quantified by gas chromatography mass spectrometry of 24 h urine samples) over time [53]. In contrast, older females on creatine (*n* = 7) experienced a small, yet significant increase in 3-MH. Finally, there was a reduction in 3-MH (analyzed with high-performance liquid chromatography of 24 h urine samples) after 12 weeks of whole-body resistance training in older adults supplementing with creatine (0.1 g∙kg∙d^−1^ or ~9 g∙d^−1^; *n* = 22) either before or after training sessions [54]. Similar to the Cornish et al. study [51], no sex analysis on differences in 3-MH over time were performed. The results of the Candow et al. study was further limited as no placebo (control) group was incorporated in the study design [54]. 

As a secondary indicator of muscle protein catabolism, a recent systematic review and meta-analysis involving 278 participants (20–60 years of age) concluded that creatine supplementation had essentially no effect on measures of exercise-induced muscle damage (i.e., creatine kinase, lactate dehydrogenase) [55]. Furthermore, regarding the potential synergistic effect of creatine and other purported compounds, which may exhibit anti-catabolic properties) [56] showed no significant effect from the combination of creatine supplementation (3–10 g∙d^−1^) and β-hydroxy β-methylbutyrate (HMB; 3 g∙d^−1^) on measures of exercise-induced muscle damage (i.e., creatine kinase, lactate dehydrogenase) or catabolic hormones (i.e., cortisol) in males (*n* = 201) primarily engaged in intermittent teams sports (i.e., rugby basketball, soccer). Collectively, creatine supplementation, alone or in combination with HMB, does not appear to be a viable strategy to consistently attenuate exercise-induced muscle damage. 

In summary, results across studies indicate that creatine supplementation can decrease some measures of muscle protein catabolism, with and without resistance training, primarily in males. While speculative, the lack of effect from creatine in females may be related to intramuscular baseline (pre-supplementation) creatine levels. There is some evidence to indicate that females (when compared to males) have higher resting muscle creatine levels [57] and, therefore, may not respond as well to creatine supplementation over time [53]. It is important to highlight that in all the studies discussed above that showed a decrease in 3-MH from creatine (~9 g∙d^−1^) and resistance training over time, there was also a corresponding increase in measures of muscle mass and/or strength. Therefore, it is possible that the anti-catabolic effects from creatine on muscle protein (as measured by 3-MH) created a more favorable environment for muscle accretion and strength gains over time. Creatine supplementation has no meaningful effect on measures of exercise-induced muscle damage. 

In addition to the anti-catabolic effects of creatine on muscle, there is some evidence to suggest that creatine supplementation during a resistance training program decreases measures of bone catabolism, which may influence the bone remodeling process. Healthy older males who supplemented with creatine (0.1 g∙kg∙d^−1^ or ~9 g∙d^−1^; *n* = 23) during 10 weeks of supervised, whole-body resistance training (3 d∙wk) experienced a significant reduction in the urinary excretion of cross-linked N-telopeptides of Type I collagen (NTx; indicator of bone catabolism), quantified by competitive-inhibition ELISA, by ~25% compared to an increase (~10%) for those on placebo (*n* = 12) [52]. In healthy younger adults, 5 weeks of creatine supplementation (*n* = 47) and resistance training (6 d∙wk) resulted in a greater reduction, albeit non-significant (*p* = 0.055), in NTx (measured by competitive-inhibition ELISA of urine) compared to those on protein only (*n* = 22) [51]. These potential anti-catabolic effects from creatine on bone may help explain the reduction in bone mineral density loss (femoral neck), determined with dual-energy X-ray absorptiometry in array mode, in healthy postmenopausal females after 1 year of creatine supplementation (0.1 g∙kg∙d^−1^; *n* = 15) and supervised, whole-body resistance training compared to placebo (*n* = 18) [58]. From a clinical perspective, young boys with muscular dystrophy (*n* = 5) who supplemented with creatine (3 g∙d^−1^) for 12 weeks experienced a 56% decrease in bone collagen equivalents (indicator of NTx by quantified by competitive-inhibition ELISA) compared to a 6% increase for those on placebo [59]. Furthermore, Tarnopplsky et al. [60] subsequently showed that creatine supplementation (0.1 g∙kg∙d^−1^) decreased NTx by 22% in young boys with muscular dystrophy. In contrast to these positive findings, Gualano et al. found no effect from creatine supplementation (loading phase: 20 g∙d^−1^ for 5 d; maintenance phase: 5 g∙d^−1^ for an additional 24 wks) and supervised whole-body resistance training (*n* = 15) on changes in Type 1 collagen C-telopeptide (indicator of bone resorption; serum concentrations were determined with an automated Roche electrochemiluminescence system) compared to placebo (*n* = 15) in older females [61]. Independent of resistance training, very low-dosage creatine (1 g∙d^−1^ for 1 year, *n* = 56; or 3 g∙d^−1^ for 2 years, *n* = 106) had no effect on measures CTX (serum analysis with automated Roche electrochemiluminescence system) compared to placebo (*n* = 53 and *n* = 94) [62,63]. Results across studies provided some evidence that creatine supplementation (~9 g∙d^−1^) can attenuate bone catabolism (primarily in older adults), but only when combined with resistance training. While speculative, the decrease in measures of bone catabolism may be related to creatine influencing the bone remodeling process. For example, there is some evidence that creatine can increase osteoblast cell activity [64], which may subsequently increase osteoprotegerin and attenuate osteoclast differentiation [65]. Alternatively, increased muscle accretion from creatine may act as a pulley and bone as a lever during muscle contractions resulting in osteocyte-induced bone formation [9]. Future research is needed to determine the long-term mechanistic effects of creatine, with and without resistance training, on bone microarchitecture.

## 4. Conclusions

**Figure 1:**
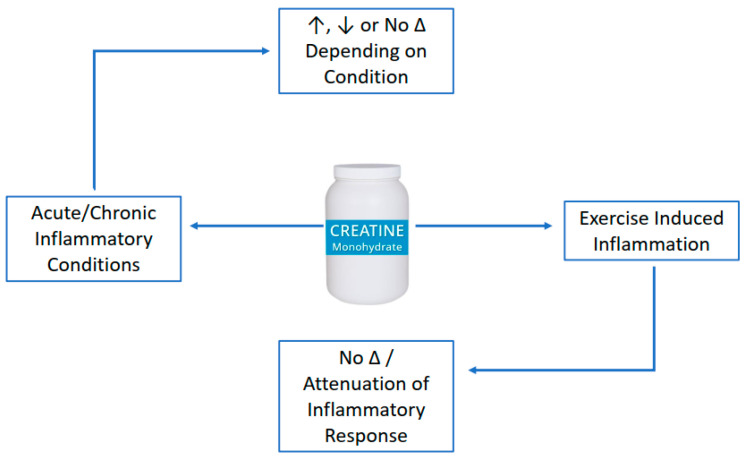
Anti-inflammatory of creatine supplementation.

**Figure 2:**
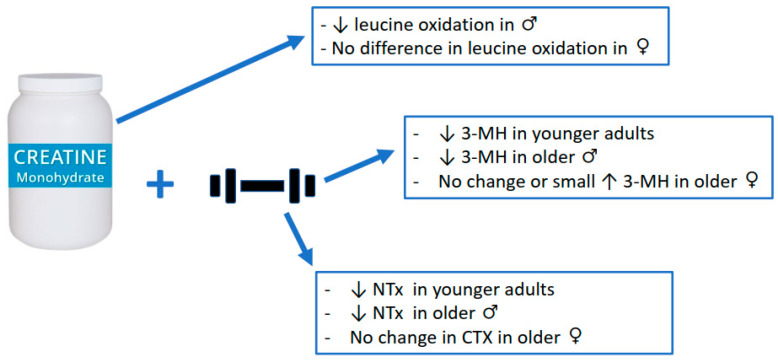
Anti-catabolic effects of creatine supplementation.

It is well established that creatine supplementation has favorable effects on muscle and bone, primarily when combined with an exercise-training stimulus. Accumulating evidence across a variety of research populations indicates that creatine supplementation has the ability to provide anti-inflammatory (Figure 1) and anti-catabolic effects (Figure 2). These findings may have application for athletes/exercising individuals, disease-state populations characterized by elevated inflammation (i.e., type II diabetes), and older adults prone to muscle (i.e., sarcopenia) and bone loss (i.e., osteoporosis). The number of studies examining the anti-inflammatory effects of creatine supplementation in various disease and exercise states continues to grow with promising results. The current evidence suggests that creatine can decrease markers of inflammation in disease or following a bout of exercise, but the response may be species and model-specific. More mechanistic-based research is needed to identify the potential anti-inflammatory effects of creatine supplementation. Further, longer-term studies that evaluate the effects of creatine supplementation on various inflammatory diseases are necessary to ascertain the putative anti-inflammatory effects of this nutritional supplement. While the body of literature examining the anti-catabolic effects of creatine supplementation is small, there is some evidence to suggest that creatine decreases some measures of muscle protein breakdown (primarily in males) and bone catabolism. Long-term, randomized controlled trials are needed to determine the mechanisms explaining the possible sex-related differences in muscle protein kinetics from creatine supplementation, with and without a resistance training intervention.

## Data Availability

Not applicable.
